# Impact of underlying heart disease per se on the utility of preoperative NT-proBNP in adult cardiac surgery

**DOI:** 10.1371/journal.pone.0192503

**Published:** 2018-02-08

**Authors:** Huiqi Jiang, Henrik Hultkvist, Jonas Holm, Farkas Vanky, Yanqi Yang, Rolf Svedjeholm

**Affiliations:** 1 Department of Cardiothoracic Surgery and Cardiothoracic Anesthesia, Faculty of Medicine and Health Sciences, Division of Cardiovascular Medicine, Linköping University, Linköping, Sweden; 2 Department of Cardiothoracic Surgery, Sun Yat-sen Memorial Hospital, Sun Yat-sen University, Guangzhou, Guangdong, China; Scuola Superiore Sant'Anna, ITALY

## Abstract

**Objective:**

The primary aim was to investigate the role of underlying heart disease on preoperative NT-proBNP levels in patients admitted for adult cardiac surgery, after adjusting for the known confounders age, gender, obesity and renal function. The second aim was to investigate the predictive value of preoperative NT-proBNP with regard to severe postoperative heart failure (SPHF) and postoperative mortality.

**Methods:**

A retrospective cohort study based on preoperative NT-proBNP measurements in an unselected cohort including all patients undergoing first time surgery for coronary artery disease (CAD; n = 2226), aortic stenosis (AS; n = 406) or mitral regurgitation (MR; n = 346) from April 2010 to August 2016 in the southeast region of Sweden (n = 2978). Concomitant procedures were not included, with the exception of Maze or tricuspid valve procedures.

**Results:**

Preoperative NT-proBNP was 1.67 times (p<0.0001) and 1.41 times (p<0.0001) higher in patients with AS or MR respectively, than in patients with CAD after adjusting for confounders.

NT-proBNP demonstrated significant discrimination with regard to SPHF in CAD (AUC = 0.79, 95%CI 0.73–0.85, p<0.0001), MR (AUC = 0.80, 95%CI 0.72–0.87, p<0.0001) and AS (AUC = 0.66, 95%CI 0.51–0.81, p = 0.047). In CAD patients NT-proBNP demonstrated significant discrimination with regard to postoperative 30-day or in-hospital mortality (AUC = 0.78; 95%CI 0.71–0.85, p<0.0001). The number of deaths was too few in the AS and MR group to permit analysis. Elevated NT-proBNP emerged as an independent risk factor for SPHF, and postoperative mortality in CAD.

**Conclusions:**

Patients with AS or MR have higher preoperative NT-proBNP than CAD patients even after adjusting for confounders. The predictive value of NT-proBNP with regard to SPHF was confirmed in CAD and MR patients but was less convincing in AS patients.

## Introduction

Postoperative heart failure (PHF) remains the major cause of mortality after cardiac surgery [1–6]. The most recent international guidelines recommend the use of natriuretic peptides, particularly BNP or NT-proBNP, as first-line biomarkers for the diagnosis, prognosis, and follow-up of patients with heart failure[7, 8]. In adult patients undergoing cardiac surgery elevated preoperative natriuretic peptide levels are associated with postoperative heart failure and postoperative mortality [9–13]. In addition, natriuretic peptides may be useful in pediatric patients undergoing cardiac surgery[14]. The impact of underlying heart disease per se on preoperative NT-proBNP levels has not been fully clarified. Furthermore, it is not known if the association between preoperative NT-proBNP and postoperative heart failure and early outcome differs in the patients with coronary artery disease (CAD), aortic stenosis (AS) or mitral valve regurgitation (MR).

A limited number of studies have reported that preoperative natriuretic peptide levels differ between patients admitted for coronary artery bypass surgery (CABG), aortic valve replacement (AVR) and mitral valve surgery [15–17]. However, the sample size of these studies were either small or did not adjust for confounders, such as age, female gender, preoperative renal function, and obesity, which have been reported to influence natriuretic peptides[18–22].

The primary aim of this study was to investigate the impact of CAD, AS, and MR on NT-proBNP levels in patients admitted for surgery after adjusting for known confounders not directly related to the heart disease. The second aim was to investigate the predictive value of preoperative NT-proBNP in these cohorts with regard to severe postoperative heart failure (SPHF) and postoperative mortality.

## Materials and methods

### Ethics

Ethical approval (Dnr 2011/ 497–31) was provided for the study by the Regional Ethical Review Board in Linköping, Sweden. The study was performed according to the Helsinki Declaration of Human Rights. Owing to the nature of the study, the ethics committee waived the need for written informed consent.

### Patients

**Fig 1:**
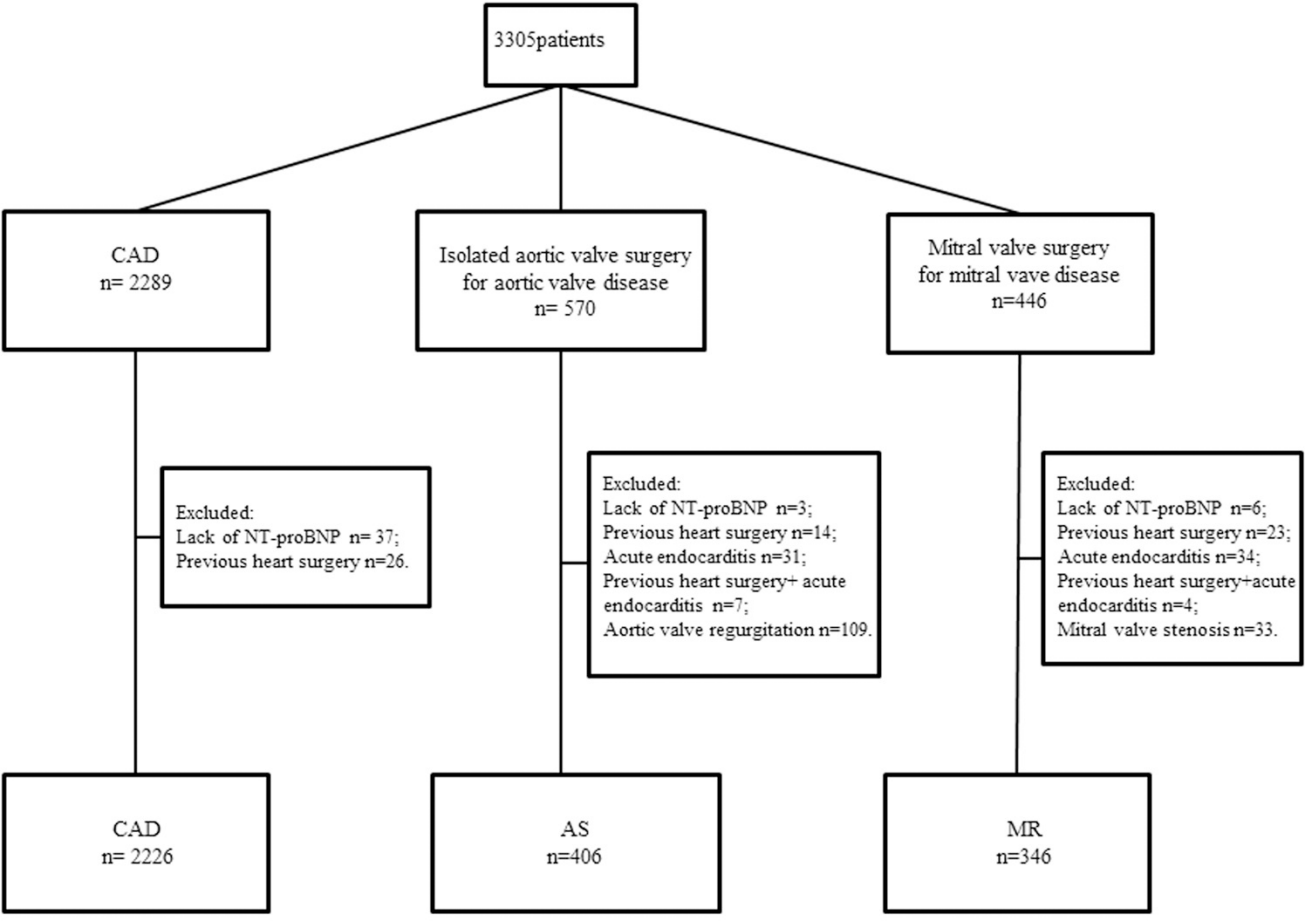
Patient flow chart. CAD: patients with coronary artery disease admitted for first time CABG, AS: patients with aortic stenosis without significant aortic regurgitation admitted for first time aortic valve replacement, MR: patients with mitral regurgitation without significant mitral stenosis admitted for first time mitral valve surgery with or without tricuspid valve procedure. A total of 161 patients underwent mitral valve surgery with tricuspid valve procedure, and, 102 patients had concomitant Maze procedure (26 patients undergoing CABG, 12 undergoing isolated aortic valve surgery, and 64 undergoing mitral valve surgery).

The University Hospital in Linköping is the only referral center in the southeast region of Sweden, serving a population of approximately 1 million. The decision to perform surgery is based on current guidelines [23, 24]. From April 30, 2010, to August 31, 2016, 4991 patients underwent cardiac surgery at this department. From this cohort, we included 2978 consecutive patients admitted for first time isolated CABG for CAD (n = 2226), isolated AVR because of AS without significant aortic regurgitation (n = 406) or mitral valve surgery due to MR without mitral valve stenosis (n = 346). Concomitant Maze or tricuspid valve procedures were not exclusion criteria. Patients undergoing redo procedures (n = 63), patients with acute endocarditis (n = 65), and patients without preoperative NT-proBNP values (n = 46) were excluded. Eleven patients were excluded because of endocarditis after previous surgery (Fig 1).

### Study design

This was a retrospective cohort study. NT-proBNP was routinely measured the day before surgery in elective patients and on the day of surgery in emergency patients. Blood samples were collected in lithium heparin tubes and analyzed within 1 hour (emergency) to 3 hours (elective patients). NT-proBNP was measured in plasma by electro-chemoiluminescence immunoassay on a Roche Elecsys 2010 automated platform (Roche Diagnostics, Basel, Switzerland) at Linköping University Hospital. The assay had an effective measuring range of 5–35 000 ng/L. The inter-assay coefficient of variation was at 175 ng/L CV = 2.7%, 355 ng/l CV = 2.4% and 1068 ng/L CV = 1.9%. The following upper reference limits (URLs) were applied: 450 ng/L for <50 years, 900 ng/L for 50–75 years, and 1800 ng/L for >75 years. Values < 300 ng/L were considered normal in all age groups and the intervals between 300 ng/Land the URL for the age group were considered a grey zone [25, 26].

### Data collection

Demographic and perioperative data were registered prospectively in a computerized institutional database (Carath version 5.4, Fujitsu Inc.). All fields were defined in a data dictionary. The variables included in our analysis are shown in the supplementary data (S1 Table). With regard to postoperative mortality, the follow-up time was 90 days. Mortality data were retrieved from the Swedish Civil registry. The cause of death was retrieved from medical records and usually supported by autopsy.

### Study endpoints

The primary endpoint was preoperative NT-proBNP level in CAD, AS and MR patients after adjusting for the known confounders age, gender, obesity and renal function. The secondary endpoint was the predictive value of preoperative NT-proBNP in CAD, AS and MR patients with regard to SPHF and postoperative 30-day or in-hospital mortality.

### Definitions

Emergency operation was defined as a procedure that could not be postponed until the following day and, therefore, was usually performed immediately but not later than 24 hours from acceptance.

Preoperative left ventricular (LV) systolic function was assessed by cardiologists dedicated to echocardiography and categorized as normal, mildly depressed, moderately depressed, or severely depressed systolic LV function. Moderate or severe LV dysfunction corresponds to a LV ejection fraction of ≤ 45%. Severe LV dysfunction corresponds to a LV ejection fraction ≤ 30%.

Preoperative congestive heart failure was considered in patients with ongoing pharmacological treatment for heart failure who received the diagnosis from a cardiologist.

Pulmonary hypertension was defined by a systolic pulmonary artery pressure > 60 mm Hg.

Critical preoperative state was defined as ventricular tachycardia, ventricular fibrillation, or cardiopulmonary resuscitation; the need for preoperative ventilator treatment before admission to the operating room; need for preoperative inotropes or IABP; or preoperative acute renal failure (anuria or oliguria < 10ml/h).

Patients were considered to have SPHF if supported by clinical diagnosis in the medical records and/or echocardiographic signs of heart failure and an ICU stay ≥ 72 hours or hospital mortality with one of the following: intra-aortic balloon pump or ventricular assist device, or the use of inotropes (adrenaline ≥ 3 μg/min; milrinone ≥ 0.375 μg kg^-1^ min^-1^; need for two inotropes at any dosage; or use of levosimendan at any dosage).

Postoperative mortality was defined as the rate of death from any cause within 30 days after cardiac surgery, or death from any cause later during the same hospitalization period, including discharge to the referral hospital. Medical records were scrutinized for all patients dying within 90 days of surgery.

Postoperative stroke was defined as a focal neurological deficit persisting for more than 24 hours or depression of consciousness or confusion if associated with signs of cerebral injury on CT-scan. Cognitive dysfunction was not assessed.

### Statistical analysis

Data are presented as percentages or mean ± standard deviation or median (interquartile range). The Mann-Whitney U test was used to compare continuous variables between two groups. The Kruskal-Wallis test and pairwise comparison test were used to compare continuous variables among patients with CAD, AS and MR. The Pearson Chi-square test was used to compare proportions and the Bonferroni correction was used to account for multiple testing. Preoperative NT-proBNP was log_10_ transformed before linear regression analysis because of its skewed distribution.

To assess the role of underlying heart disease (i.e. diagnosis of CAD, AS, or MR) on NT-proBNP levels at admission to surgery, a multivariable linear regression model with regard to log_10_ NT-proBNP was used, adjusting for the known confounders age, renal function, gender and obesity. A multivariable linear regression model was also used to assess the role of underlying heart disease with regard to log_10_ NT-proBNP, taking into account both the diagnosis and all available data, including the consequences of the disease. Finally, a multivariable linear regression model was used separately in patients with CAD, AS, and MR to determine variables independently associated with log_10_ NT-proBNP within these diagnostic groups. Potential variables were assessed by guidance of univariate analysis (p<0.25). Collinearity diagnostics were performed using variance inflation factors to exclude unacceptable collinearity between selected variables.

A receiver operating characteristic (ROC) analysis was performed to calculate the area under the curve (AUC) in order to evaluate the discrimination of preoperative NT-proBNP with regard to SPHF and postoperative mortality. Youden´s index was used to calculate the best cutoff point with regard to sensitivity and specificity. Furthermore, the cutoff point providing a similar sensitivity and specificity in each diagnostic group at or just above 75% was calculated. Univariate preoperative and intra-operative predictors of SPHF and postoperative mortality were analyzed using the Mann-Whitney U-test for non-parametrically distributed continuous data and the Pearson chi-squared test for nominal data. All variables with p < 0.25 were entered into a backward (conditional) stepwise multivariable logistic regression to identify preoperative and intra-operative risk factors for SPHF and postoperative mortality. Hosmer-Lemeshow goodness-of-fit statistics were calculated for the final model. Statistical significance was defined as p < 0.05. Statistical analyses were performed in SPSS statistics version 23 (IBM) for Windows and Statistica 13.2 (Dell Inc.).

## Results

### Patient characteristics

Among all 2978 patients, the median age was 70 [63–76] years and 24% were female. The median Additive EuroSCORE was 4[3–6]. Age, preoperative estimated glomerular filtration rate (eGFR), hemoglobin, albumin, proportion of moderate or severe LV dysfunction, atrial fibrillation, and obesity were significantly different among patients with CAD, AS, and MR. Detailed preoperative, intraoperative, and postoperative characteristics are given in Tables 1 and 2. Data for the patients (n = 46) who were excluded because NT-proBNP was not sampled before surgery are given in the supplementary data (S2 Table). These patients had a similar additive EuroSCORE but a higher proportion of emergency operations and NYHA IV, and higher postoperative mortality than the study cohort.

**Table 1:** Demographic data in all patients and subgroups.

	All patients (n = 2978)	CAD (n = 2226)	AS (n = 406)	MR (n = 346)	p*	P^†^	p^‡^	p^§^
Age (years)	70 [63–76]	69 [63–75]	73 [66–78]	67 [58–74]	<0.0001	<0.0001	<0.0001	<0.0001
Female gender	24% (714)	18% (410)	50% (202)	29% (102)	<0.0001	<0.0001	<0.0001	<0.0001
Preop-NT-proBNP (ng/L)	330 [130–970]	290 [120–833]	595 [260–1510]	400 [110–1350]	<0.0001	<0.0001	0.050	<0.0001
BMI kg/m^2^	27 [24–30]	27 [25–30]	27 [24–31]	25 [23–28]	<0.0001	1.000	<0.0001	<0.0001
Obesity (BMI ≥ 30kg/m^2^)	26% (782)	27% (606)	32% (130)	13% (46)	<0.0001	0.051	<0.0001	<0.0001
BSA m^2^	1.96 [1.83–2.09]	1.98 [1.85–2.10]	1.90 [1.77–2.05]	1.93 [1.80–2.06]	<0.0001	<0.0001	<0.0001	0.58
Preop hemoglobin (g/L)	141 [132–150]	141 [132–150]	140 [130–148]	142 [133–149]	0.024	0.025	1.000	0.08
Preop p-creatinine (μmol/L)	85 [74–101]	86 [74–102]	80 [68–97]	83 [73–98]	<0.0001	<0.0001	0.07	0.11
Preop-eGFR mL•min^-1^ •1.73m^-2^	73 [60–86]	73 [60–86]	72 [58–86]	75 [62–87]	0.29	0.47	0.20	0.13
Preop p-albumin (g/L)	39 [36–42]	39 [36–42]	40 [37–42]	40 [38–43]	<0.0001	<0.0001	<0.0001	1.0
Smoker	11% (336)	13% (299)	5% (22)	4% (15)	<0.0001	<0.0001	<0.0001	0.50
Diabetes	25% (739)	29% (655)	17% (67)	5% (17)	<0.0001	<0.0001	<0.0001	<0.0001
Hypertension	65% (1934)	70% (1558)	59% (240)	39% (136)	<0.0001	<0.0001	<0.0001	<0.0001
COPD	7% (219)	8% (173)	6% (23)	7% (23)	0.28	0.14	0.46	0.58
Cerebrovascular disease	7% (208)	8% (167)	5% (19)	6% (22)	0.28	0.041	0.45	0.31
Extracardiac arterial disease	7% (208)	8% (187)	4% (15)	2% (6)	<0.0001	0.001	<0.0001	0.10
Previous vascular surgery	4% (115)	5% (101)	2% (9)	1% (5)	0.004	0.031	0.007	0.44
Preop dialysis	0.8% (25)	0.8% (18)	1.3% (5)	0.6% (2)	0.59	0.39	0.63	0.46
Angina	74% (2207)	97% (2151)	14% (56)	0	<0.0001	<0.0001	<0.0001	<0.0001
Unstable angina	46% (1372)	62% (1372)	0	0	<0.0001	<0.0001	<0.0001	-
CCS IV	9% (271)	12% (271)	0	0	<0.0001	<0.0001	<0.0001	-
Recent myocardial infarction (<3 weeks)	28% (839)	38% (835)	0.7% (3)	0.3%(1)	<0.0001	<0.0001	<0.0001	0.40
Moderate or severe LV dysfunction	17% (501)	19% (415)	12% (49)	11% (37)	<0.0001	0.001	<0.0001	0.56
Severe LV dysfunction	4% (134)	5% (121)	2% (10)	0.9% (3)	<0.0001	0.011	<0.0001	0.09
Preop pulmonary hypertension	3% (81)	1% (21)	2% (9)	15% (51)	<0.0001	0.028	<0.0001	<0.0001
NYHA III or IV	58% (1718)	62% (1374)	50% (203)	41% (141)	<0.0001	<0.0001	<0.0001	0.011
NYHA IV	8% (239)	9% (209)	1% (4)	8% (26)	<0.0001	<0.0001	0.26	<0.0001
Preop CHF	17% (506)	14% (306)	15% (62)	40% (138)	<0.0001	0.42	<0.0001	<0.0001
Preop atrial fibrillation	6% (187)	3% (76)	8% (31)	23% (80)	<0.0001	<0.0001	<0.0001	<0.0001
Additive EuroSCORE	4 [3–6]	4 [2–6]	6 [4–7]	5 [3–7]	<0.0001	<0.0001	<0.0001	0.07

Data given as medians [interquartile range] or percentages (number).

* among three groups

^†^ CAD vs AS

^‡^ CAD vs MR

^§^ AS vs MR.

AS: aortic stenosis, BMI: body mass index, CAD: coronary artery disease, CHF: congestive heart failure, COPD: chronic obstructive pulmonary disease, eGFR: estimated glomerular filtration rate according to MDRD formula, EuroSCORE: European system for cardiac operative risk evaluation, LV: left ventricular, MR: mitral regurgitation, NYHA: New York Heart Association functional classification.

**Table 2:** Intra- and postoperative characteristics in all patients and subgroups.

	All patients (n = 2978)	CAD (n = 2226)	AS (n = 406)	MR (n = 346)	p*	P^†^	p^‡^	p^§^
Emergency operation	3% (88)	3% (69)	0.2% (1)	5% (18)	<0.0001	0.001	0.044	<0.0001
Critical preoperative state	1% (30)	1% (19)	0.5% (2)	3% (9)	0.005	0.76	0.004	0.016
CPB time (minutes)	84 [68–104]	78 [64–95]	90 [79–105]	134 [108–166]	<0.0001	<0.0001	<0.0001	<0.0001
Aortic cross clamp time (minutes)	56 [44–70]	51 [40–62]	65 [56–75]	92 [76–116]	<0.0001	<0.0001	<0.0001	<0.0001
ICU stay (hours)	21 [18–23]	21 [17–23]	22 [18–23]	21 [20–23]	<0.0001	0.048	<0.0001	0.44
ICU stay >72 hours	6% (192)	6% (133)	6% (26)	10% (33)	0.043	0.74	0.012	0.11
Ventilation time (hours)	3 [2–5]	3 [2–5]	3 [2–5]	3 [2–6]	0.001	0.21	0.001	0.37
Ventilation time >48 hours	3% (100)	3% (65)	2% (8)	8% (27)	<0.0001	0.29	<0.0001	<0.0001
Severe PHF	4% (130)	4% (88)	3% (14)	8% (28)	0.001	0.63	0.001	0.006
Postoperative stroke	0.7% (22)	0.9% (19)	0.5% (2)	0.3% (1)	0.43	0.76	0.51	1.0
CK-MB POD1 (μg/L)	16 [10–28]	14 [9–23]	16 [12–23]	40 [26–68]	<0.0001	<0.0001	<0.0001	<0.0001
CK-MB POD1 >50 μg/L	12% (345)	9% (202)	5% (22)	35% (121)	<0.0001	0.012	<0.0001	<0.0001
Creatinine elevation ≥ 50%	9% (262)	8% (186)	11% (46)	9% (30)	0.16	0.054	0.83	0.24
Postoperative mortality	2% (53)	2% (43)	1% (4)	2% (6)	0.41	0.19	1.000	0.53

Data given as medians [interquartile range] or percentages (number).

* among three groups

^†^ CAD vs AS

^‡^ CAD vs MR

^§^ AS vs MR.

AS: aortic stenosis, CAD: coronary artery disease, CPB: cardiopulmonary bypass, CK-MB: creatine kinase-MB isoenzyme, ICU: intensive care unit, MR: mitral regurgitation, POD1: first postoperative day, PHF: postoperative heart failure.

### Preoperative NT-proBNP and underlying heart disease

The distribution of preoperative NT-proBNP of the whole cohort exhibited a positive skewness with a median value of 330 [130–970] ng/L. NT-proBNP was higher in patients with AS than in patients with CAD (595 [260–1510] vs 290 [120–833] ng/L, p<0.0001) or patients with MR (400 [110–1350] ng/L, p<0.0001). The proportion of patients with NT-proBNP values greater than the age-adjusted URL was higher in patients with AS (32%) or MR (30%) than in patients with CAD (20%). The proportion of patients with NT-proBNP < 300 ng/L was 50% for CAD, 43% for MR, and 30% for AS (Table 3).

**Table 3:** Proportion of patients within age-adjusted reference limits for NT-proBNP.

	CAD (n = 2226)	AS (n = 406)	MR (n = 346)	p^§^	p**	p^††^
Rule out*	50% (1119)	30% (120)	43% (150)	<0.0001	0.017	0.0001
Grey zone^†^	29% (652)	38% (156)	27% (93)	0.0002	0.36	0.0008
> Age -adjusted URL^‡^	20% (455)	32% (130)	30% (103)	<0.0001	0.0001	0.51

The number of patients is given in parentheses.

*Congestive heart failure unlikely, percentage of patients with NT-proBNP <300 ng/L.

^†^Congestive heart failure possible, percentage of patients with NT-proBNP dependent on age: between 300 and 450 ng/L for <50 years; between 300 and 900 ng/L for 50–75 years; between 300 and 1800 ng/L for >75 years.

^‡^ “Rule in” Congestive heart failure likely, percentage of patients with NT-proBNP >450 ng/L for <50 years, >900 ng/L for 50–75 years, and >1800 ng/L for >75 years.

^§^ CAD vs AS

** CAD vs MR

^††^ AS vs MR.

CAD: coronary artery disease, AS: aortic valve stenosis, MR: mitral valve regurgitation.

After adjusting for age, eGFR, female gender, and obesity, NT-proBNP was 1.67 times higher in patients with AS than in patients with CAD (adjusted coefficient 0.223, 95% CI 0.160–0.285; p<0.0001) and 1.41 times higher in patients with MR than patients with CAD (adjusted coefficient 0.150, 95% CI 0.085–0.215, p<0.0001; Table 4).

**Table 4:** Multivariable linear regression results for log_10_NTproBNP in all patients.

	Adjusted coefficient	95%CI	p
Age (years)	0.020	0.018–0.022	<0.0001
Preop eGFR (mL•min^-1^ •1.73m^-2^)	-0.006	-0.007 - -0.005	<0.0001
Male	ref		
Female	0.114	0.064–0.164	<0.0001
CAD	ref		
MR	0.150	0.085–0.215	<0.0001
AS	0.223	0.160–0.285	<0.0001

Adjusted R^2^ = 0.215, ANOVA for the model (df = 5, F = 164.162, p<0.0001) eGFR: estimated glomerular filtration rate according to MDRD formula, CAD: coronary artery disease, AS: aortic valve stenosis, MR: mitral valve regurgitation.

### Preoperative NT-proBNP and severe postoperative heart failure

A total of 130 patients had SPHF (88 patients with CAD, 14 patients with AS, 28 patients with MR). Patients with SPHF had significantly higher level of preoperative NT-proBNP than patients without SPHF in the whole cohort and in the subgroups with CAD, MR, or AS (Table 5).

**Table 5:** Preoperative NT-proBNP in patients with or without SPHF and postoperative mortality.

	SPHF			Postoperative mortality		
	Yes	No	p	Yes	No	p
All	2060[753–4910]	310[120–910]	<0.0001	1780[430–3200]	320[130–958]	<0.0001
CAD	2345[655–5810]	280[110–750]	<0.0001	1810[440–3260]	280[120–800]	<0.0001
AS	1290[563–4018]	585[250–1498]	0.047	2835[230–10090]	595[260–1510]	0.53
MR	1650[925–3802]	345[97–1115]	<0.0001	1245[430–2640]	395[110–1280]	0.08

Preoperative NT-proBNP (ng/L) given as medians [interquartile range]. SPHF: severe postoperative heart failure, All: all patients, CAD: coronary artery disease, AS: aortic valve stenosis, MR: mitral valve regurgitation.

**Fig 2:**
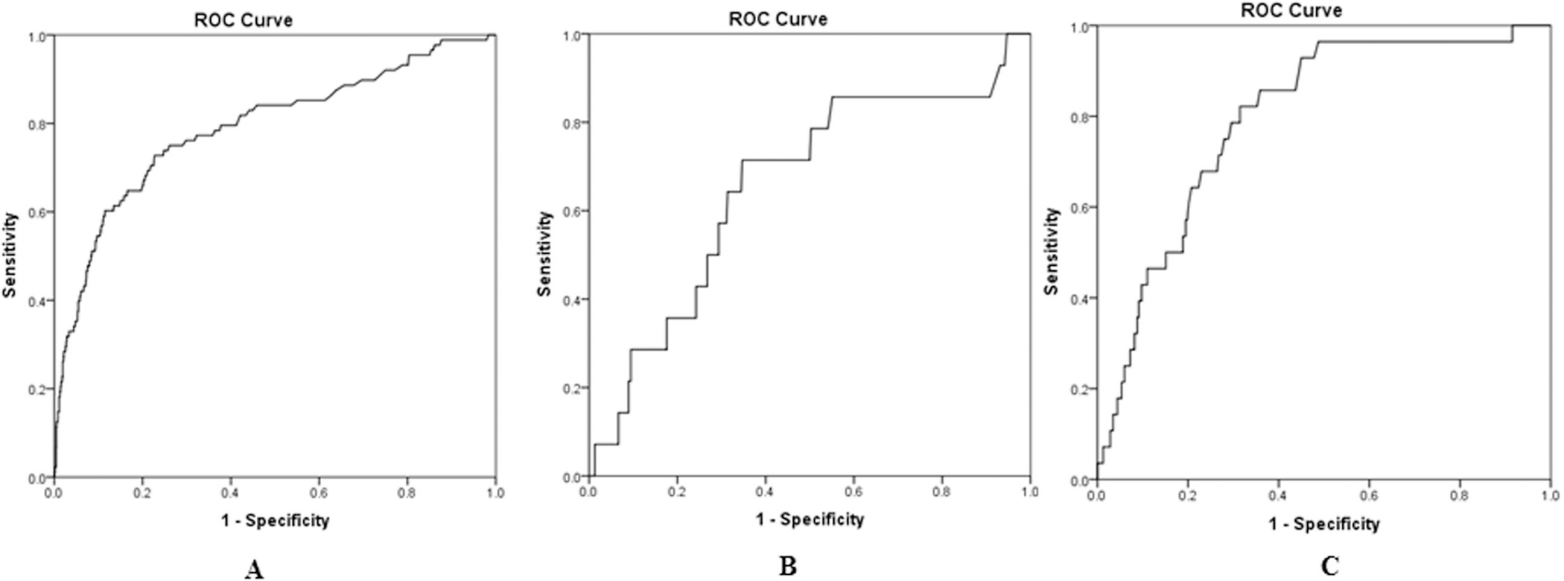
Discrimination of preoperative NT-proBNP with regard to SPHF in patients with CAD, AS, and MR. Preoperative NT-proBNP demonstrated significant discrimination according to ROC analysis with regard to SPHF in patients with CAD (A: AUC = 0.79, 95% CI 0.73–0.85, p<0.0001; best cutoff 855 ng/L with a sensitivity of 73% and a specificity of 77%), AS (B: AUC = 0.66, 95% CI 0.51–0.81, p = 0.047; best cutoff 975 ng/L with a sensitivity of 71% and specificity of 65%), and MR (C: AUC = 0.80, 95% CI 0.72–0.87, p<0.0001; best cutoff 800 ng/L with a sensitivity of 82% and specificity of 69%). ROC: receiver operating characteristics, SPHF: severe postoperative heart failure, AUC: area under the curve, CI: confidence interval.

Preoperative NT-proBNP demonstrated significant discriminatory power with regard to SPHF in patients with CAD (AUC = 0.79, 95% CI 0.73–0.85, p<0.0001), MR (AUC = 0.80, 95% CI 0.72–0.87, p<0.0001) and AS (AUC = 0.66, 95% CI 0.51–0.81, p = 0.047; Fig 2). The best cutoffs according to Youden´s index were 855 ng/L (sensitivity 73%, specificity 77%) in CAD patients, 975 ng/L (sensitivity 71%, specificity 65%) in AS patients and 800 ng/L (sensitivity 82%, specificity 69%) in MR patients (Fig 2).

To achieve more comparable cutoff values for preoperative NT-proBNP with regard to SPHF in different subgroups, sensitivity and specificity was kept at or just above 75% (Table 6). At 79% sensitivity cutoff values were fairly similar for CAD (435 ng/L) and AS patients (575 ng/L) but appeared substantially higher for MR patients (900 ng/L).

**Table 6:** Cutoffs for preoperative NT-proBNP with regard to SPHF in subgroups at equal sensitivity and specificity.

	Sensitivity	Specificity	Cutoff* (ng/L)	Sensitivity	Specificity	Cutoff^†^ (ng/L)
CAD	79.5%	62%	435	74%	75%	755
AS	79%	50%	575	43%	75%	1485
MR	79%	70%	900	68%	75%	1120

*Sensitivity was kept around 79%.

^†^Specificity was kept around 75%.

SPHF: severe postoperative heart failure, CAD: coronary artery disease, AS: aortic valve stenosis, MR: mitral valve regurgitation.

In the multivariable analysis, NT-proBNP ≥ 855 ng/L emerged as an independent risk factor for SPHF in patients with CAD (adjusted OR 2.87, 95% CI 1.56–5.30, p = 0.001). Age, preoperative dialysis, aortic cross-clamp time in upper quartile (≥ 62min), moderate to severe LV dysfunction, NYHA IV, insulin-treated diabetes, critical preoperative state, and emergency operation were the other variables in the final model (Table 7). The number of events was too few to permit multivariable analysis in patients with AS or MR.

**Table 7:** Multivariable analysis* of risk factors for SPHF in CAD patients.

Variable	Odds ratio	95% CI	p
Age (years)	1.05	1.01–1.08	0.005
Preoperative dialysis	23.1	6.47–82.2	<0.0001
Preop NT-proBNP ≥885 ng/L	2.87	1.56–5.30	0.001
Cross-clamp time upper quartile (>62 min)	3.04	1.78–5.18	<0.0001
Moderate to severe LV dysfunction	2.69	1.51–4.79	0.001
NYHA IV	2.74	1.39–5.37	0.003
Insulin-treated diabetes	2.65	1.50–4.68	0.001
Emergency operation	3.39	1.40–8.24	0.007
Critical condition preoperatively	7.49	2.19–25.7	0.001

Due to a lack of aortic cross clamp time, patients undergoing off-pump CABG are not included in this model.

*Multivariable backward stepwise logistic regression model.

Nagelkerke R^2^ = 0.322; Hosmer-Lemeshow goodness-of-fit test x^2^ (df = 8) = 7.280, p = 0.507. SPHF: severe postoperative heart failure, CAD: coronary artery disease, CI: confidence interval.

### Preoperative NT-proBNP and postoperative mortality

Fifty-three (2%) patients died postoperatively within 30 days or in-hospital; 43 due to cardiac causes, 3 due to postoperative stroke, and 6 due to other non-cardiac causes, and cause of death was unknown in 2 patients. Further details are given in the supplementary data (S3 Table). Patients with postoperative mortality had significantly higher preoperative NT-proBNP than patients without postoperative mortality (1780 [430–3200] vs 320 [130–958] ng/L, p<0.0001; Table 5).

**Fig 3:**
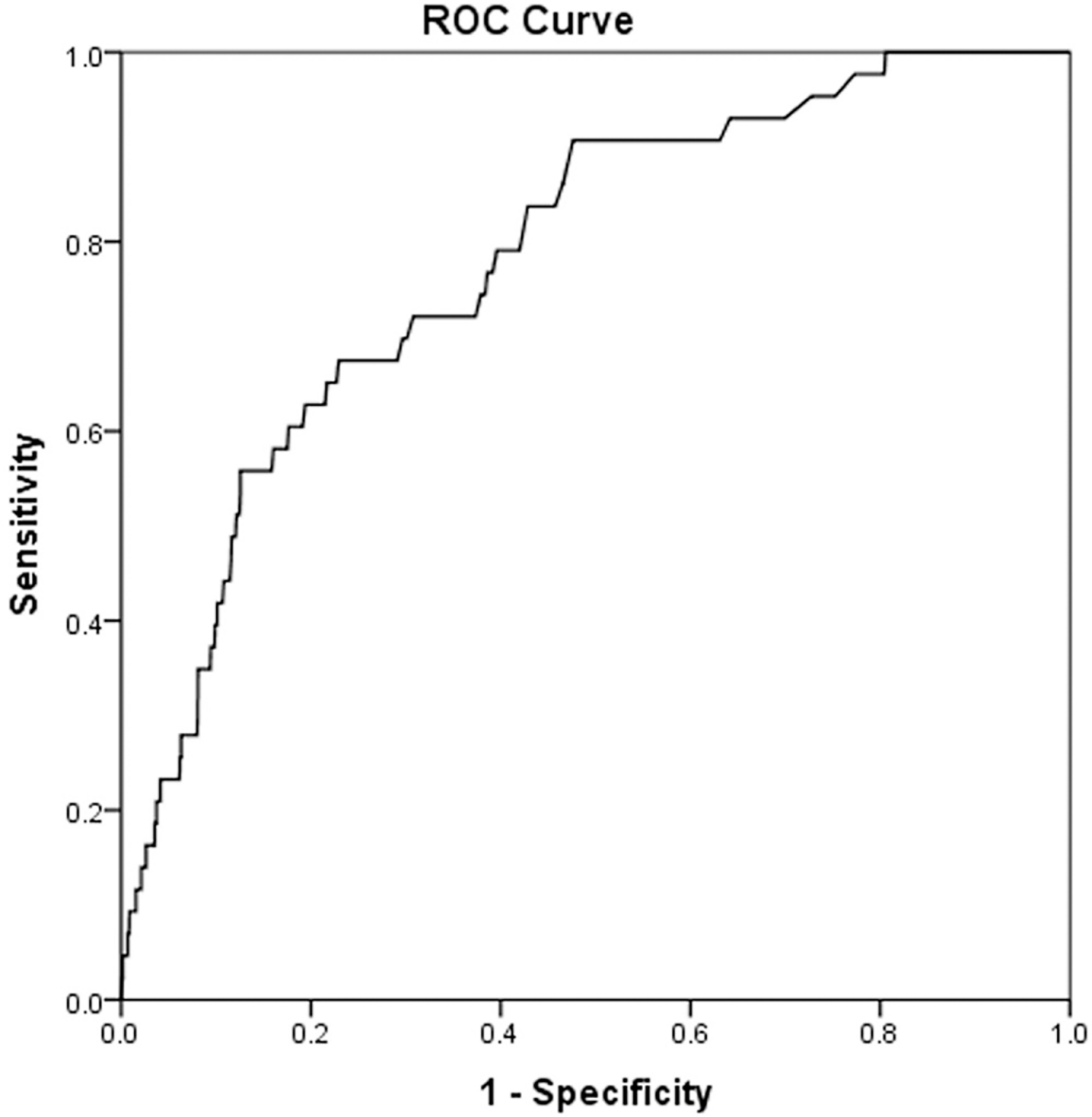
Discrimination of preoperative NT-proBNP with regard to postoperative mortality in patients with CAD. ROC analysis demonstrated an AUC of 0.78 (95% CI 0.71–0.85, p<0.0001; best cutoff 905 ng/L with a sensitivity of 67% and specificity of 77%). ROC: receiver operating characteristics, AUC: area under the curve, CI: confidence interval.

In CAD patients, preoperative NT-proBNP demonstrated significant discrimination with regard to postoperative mortality (AUC = 0.78, 95%CI 0.71–0.85, p<0.0001; best cutoff 905 ng/L with a sensitivity of 67% and specificity of 77%; Fig 3). The number of events was too few to permit ROC analysis in patients with AS (n = 4) or MR (n = 6).

NT-proBNP ≥905 ng/L emerged as an independent risk factor for postoperative mortality in patients with CAD (adjusted OR 2.56, 95% CI 1.21–5.40, p = 0.014). Age, NYHA IV, preoperative albumin, preoperative dialysis, and emergency operation also remained in the final model for postoperative mortality (Table 8).

**Table 8:** Multivariable analysis* of risk factors of postoperative mortality in CAD patients.

Variable	Odds ratio	95% CI	p
Age (years)	1.09	1.04–1.15	<0.0001
NYHA IV	2.84	1.23–6.56	0.015
Emergency operation	3.54	1.29–9.68	0.014
Preop dialysis	24.2	6.33–92.3	<0.0001
Preop NT-proBNP ≥ 905 ng/L	2.56	1.21–5.40	0.014
Preop p-albumin (g/L)	0.93	0.86–0.99	0.025

*Multivariable backward stepwise logistic regression model.

Nagelkerke R^2^ = 0.254; Hosmer-Lemeshow goodness-of-fit test x^2^ (df = 8) = 6.560, p = 0.535. CAD: coronary artery disease, CI: confidence interval, NYHA: New York Heart Association functional classification.

## Discussion

The main and novel finding of this study was that the level of preoperative NT-proBNP in patients with AS or MR was higher than in CAD patients, even after adjusting for confounders. In addition, the predictive value of preoperative NT-proBNP with regard to SPHF was good in CAD and MR patients but less convincing in AS patients. In CAD patients, NT-proBNP had good predictive value with regard to postoperative mortality, whereas the number of deaths was too few to permit analysis in AS and MR. Furthermore, preoperative NT-proBNP emerged as an independent risk factor for SPHF and postoperative mortality in CAD patients.

The primary aim of this study was to determine the impact of underlying heart disease (CAD, AS, or MR) per se on preoperative NT-proBNP levels in patients admitted for cardiac surgery. Among the studied groups, patients with AS had the highest level of preoperative NT-proBNP, whereas patients with CAD had the lowest values. These findings match those of previous studies [15–17, 27–31]. Natriuretic peptides are released from the myocardium as a response to ventricular wall stress mainly caused by volume expansion, pressure overload or ischemia [32]. However, there are also confounders that are not necessarily directly related to heart disease that could influence the level of preoperative NT-proBNP, such as age[18], female gender[18], renal function[19, 20], and obesity[33]. Increasing age, female gender, and renal dysfunction are associated with increased natriuretic peptide levels, regardless of whether the patient has heart failure, whereas obesity is associated with an inverse relationship between BMI and natriuretic peptide levels[33]. After adjusting for these confounders, AS and MR patients still had higher NT-proBNP levels than CAD patients. The results suggest that ventricular wall stress caused by pressure overload or volume overload are more important than ischemia per se for the release of natriuretic peptides. On the other hand, a substantial proportion of patients in all subgroups, and particularly the CAD group, had NT-proBNP levels < 300 ng/L at admission to surgery. The age-dependent reference limits for NT-proBNP have been designed to “rule out” or “rule in” patients with regard to congestive heart failure[26]. Ischemia is a well-documented stimulus for the release of natriuretic peptides, though it remains to be clarified to what extent this is elicited by ischemia per se or local tissue stunning[34]. Even within the “normal range” patients with CAD have higher NT-proBNP levels than those without CAD[35]. Furthermore, an increasing extent of CAD and clinical severity of CAD is associated with increasing levels of natriuretic peptides even in the absence of LV dysfunction[35–37].

The link between natriuretic peptides and valvular heart disease has received more attention as echocardiographic findings are easier to associate with natriuretic peptide levels. In MR patients, BNP reflects the hemodynamic consequences of MR, rather than the severity of MR itself [38]. In asymptomatic MR patients with preserved LV ejection fraction, longitudinal myocardial function and left atrial volume are the main determinants of BNP levels[39]. Similarly, in AS patients with preserved LV ejection fraction BNP levels reflect the echocardiographic and clinical consequences of the afterload burden on the left ventricle rather than the severity of aortic stenosis per se[40].

Several studies have shown that high levels of preoperative natriuretic peptides are associated with postoperative heart failure, need for inotropic support, in-hospital cardiac events, and postoperative mortality after cardiac surgery [9–13]. The present study adds to the evidence in CAD patients and MR patients. The discrimination of preoperative NT-proBNP with regard to SPHF was good in CAD and MR patients. The cutoff point for MR patients appeared to be somewhat higher than in CAD patients for a comparable sensitivity or specificity. In CAD patients, NT-proBNP also emerged an independent risk factor for SPHF and postoperative mortality.

The predictive value of preoperative NT-proBNP with regard to SPHF was less convincing in AS patients. This finding does not support the previous research by Fellahi et al on the prognostic utility of BNP in predicting major adverse cardiac events (MACEs), that apart from heart failure included malignant ventricular arrhythmias, Q-wave infarction, and repeat revascularization, during the first 12 months following CABG or AVR. Fellahi et al reported that preoperative BNP was accurate in predicting MACEs after AVR but not after CABG[15]. Possible explanations for the conflicting results are differences regarding endpoints, follow-up times and sample sizes. Although our sample size was larger, the incidence of SPHF among our AS patients was low. In our experience, a substantial proportion of PHF in AS patients occurs unexpectedly due to intraoperatively acquired myocardial depression or injury in low risk patients [6]. Further studies are needed to establish the predictive value of NT-proBNP in patients undergoing surgery for AS.

ROC analysis revealed a best cutoff for preoperative NT-proBNP of 905 ng/L with regard to postoperative mortality in patients with CAD. This finding is in agreement with a prospective study from our institution on a smaller sample (n = 366) undergoing isolated CABG for acute coronary syndrome, which found the best cutoff for hospital mortality to be 1028 ng/L [12]. Schachner et al reported a lower preoperative NT-proBNP cutoff level of 430 ng/L for hospital mortality in a retrospective study of 819 patients undergoing isolated CABG[41]. Apart from sensitivity and specificity, the cutoffs are likely to be influenced by mortality rate and the proportion of deaths caused by cardiac causes. In our study, cardiac causes accounted for more than 80% of hospital mortality and the cutoff achieved higher sensitivity and specificity than that reported by Schachner.

The indications and timing of surgery will obviously influence the level of natriuretic peptides. In Sweden health care is available for everyone regardless of the patient’s financial situation and the availability of cardiac surgical resources permits most patients to be treated according to current guidelines [23, 24]. This study included almost all patients with CAD, AS, or MR within an area of one million inhabitants who underwent surgery during a five-year period in southeastern Sweden. Therefore, no referral selection bias should be present. On the other hand, although sampling for NT-proBNP was 98% complete, the small proportion lacking NT-proBNP data may have contributed to a mild selection bias due to a higher proportion of emergency cases and poorer clinical outcome in these patients.

The major limitations of this study are its retrospective nature and that analyses were limited by the data available in our database. For example, data on confounders such as inflammation and pharmacological treatment were unavailable [33, 42]. On the other hand, patients with acute endocarditis were excluded. The number of events in AS and MR patients was too few to permit meaningful analyses with regard to postoperative mortality. Our attempt to achieve pure study cohorts of isolated CAD, AS and MR can be questioned. As tricuspid regurgitation was common in the MR group we decided not to exclude patients having tricuspid valve surgery in the MR group. We also decided to treat atrial fibrillation as a potential consequence of the underlying heart disease in all subgroups and, hence, a concomitant Maze procedure did not render exclusion.

### Conclusion

Patients with AS or MR admitted for first time cardiac surgery have higher preoperative NT-proBNP levels than CAD patients, even after adjusting for confounders. The predictive value with regard to severe postoperative heart failure was good in CAD and MR patients, but less convincing in AS patients. The predictive value of NT-proBNP with regard to postoperative mortality was confirmed in CAD patients whereas the number of events was too few in the other subgroups.

## Supporting information

S1 Table Completeness and availability of data for the study. (DOCX)

S2 Table Perioperative characteristics in all patients with NT-proBNP and those excluded due to missing NT-proBNP values. (DOCX)

S3 Table Causes of postoperative mortality. (DOCX)
