# Supplementary material for: Impact of underlying heart disease per se on the utility of preoperative NT-proBNP in adult cardiac surgery
Source: PLoS One. 2018 Feb 8;13(2):e0192503. doi: 10.1371/journal.pone.0192503 (PMC5805306; doi:10.1371/journal.pone.0192503)
Supplement: S1 Table — (DOCX) [file pone.0192503.s001.docx]

**S1 Table.** Completeness and availability of data for the study

| **Variable** | **completeness** |
| --- | --- |
| **Preoperative** |  |
| Name of patient | 100% |
| Personal identification number | 100% |
| Birth date | 100% |
| Age | 100% |
| Gender | 100% |
| Weight | 100% |
| Height | 100% |
| BMI | 100% |
| BSA | 98% |
| p-NT-proBNP | 100% |
| p-Hemoglobin | 100% |
| p-Creatinine | 100% |
| p-Albumin | 98% |
| Smoking | 100% |
| Diabetes / type | 100% |
| Hypertension | 98% |
| COPD | 100% |
| Cerebrovascular disease | 98% |
| Neurological dysfunction | 98% |
| Extra-cardiac arterial disease | 98% |
| Previous vascular surgery | 100% |
| Dialysis | 98% |
| Angina / type | 100% |
| CCS class | 100% |
| Preoperative intravenous nitroglycerin | 98% |
| Antiplatelet drugs used | 90% |
| Anticoagulants used | 98% |
| Left Main stenosis | 97% |
| Time of last Myocardial infarction | 98% |
| Left ventricular systolic function (echocardiography) | 100% |
| Pulmonary hypertension | 98% |
| NYHA class | 100% |
| Congestive Heart failure | 98% |
| Preoperative Atrial Fibrillation | 100% |
| Previous heart surgery | 100% |
| Time of last heart surgery | 100% |
| Previous heart surgery with CPB | 98% |
| Previous heart surgery without CPB | 98% |
| Previous valve surgery | 98% |
| Previous CABG | 98% |
| Number of previous heart surgeries | 100% |
| Emergency operation (EuroSCORE definition) | 98% |
| Other procedure than isolated CABG | 98% |
| Surgery on thoracic aorta | 98% |
| Endocarditis | 100% |
| Post Infarct VSD | 98% |
| Preoperative inotropic treatment | 98% |
| Critical condition preoperatively | 98% |
| Additive EuroSCORE | 98% |
| **Intraoperative** |  |
| Procedure code (ICD-10) | 100% |
| Diagnosis code (ICD-10) | 100% |
| Operation date | 100% |
| Name of surgeon | 100% |
| Name of anesthesiologist | 100% |
| Operation time | 100% |
| CPB time | 99% |
| Aortic cross-clamp time | 98% |
| Details of procedure | 100% |
| Aortic valve | 100% |
| Aortic valve procedure | 100% |
| Aortic valve type | 100% |
| Aortic valve size | 100% |
| Mitral valve | 100% |
| Mitral valve procedure | 100% |
| Mitral valve type | 100% |
| Mitral valve size | 100% |
| Tricuspid valve | 100% |
| Tricuspid valve procedure | 100% |
| Tricuspid valve type | 100% |
| Tricuspid valve size | 100% |
| CABG | 100% |
| Number of bypassed vessels | 100% |
| Graft material | 100% |
| Aortic scanning finding | 100% |
| Aortic dissection | 100% |
| Aortic procedure | 100% |
| Surgery on septum / free wall | 100% |
| Other cardiac procedure | 100% |
| Urgent / Emergent procedure | 100% |
| Circulation support | 100% |
| Antegrade cardioplegia | 100% |
| Retrograde cardioplegia | 100% |
| Intraoperative Inotrope use | 98% |
| Adrenaline | 98% |
| Dopamine | 98% |
| Dobutamine | 98% |
| Dopexamine | 98% |
| Milrinone | 98% |
| Levosimendan | 98% |
| Intraoperative vasoactive drug use |  |
| Phenylephrine | 98% |
| Nitroglycerine | 98% |
| Nitroprusside | 98% |
| Noradrenaline | 98% |
| Vasopressine | 98% |
| Hemodynamic status of patient at the end of operation | 98% |
| LV Failure - clinical judgement by anesthesiologist | 98% |
| RV Failure - clinical judgement by anesthesiologist | 98% |
| PAD≥24mmHg | 98% |
| SvO_2_<60% | 98% |
| SvO_2_ at weaning from CPB | 98% |
| Metabolic Support | 98% |
| Ventricular assist device (type) | 100% |
| Ventricular assist device (duration) | 100% |
| **Postoperative** |  |
| SVO_2_ on admission to ICU | 90% |
| ICU stay time | 100% |
| Number of ICU stays | 100% |
| Discharged from ICU to where | 100% |
| Ventilation time | 100% |
| ICU Inotrope use | 95% |
| Adrenaline | 95% |
| Corotrop | 95% |
| Dopamine | 95% |
| Levosimendan | 95% |
| New appearance Atrial fibrillation | 95% |
| New appearance neurological deficit | 95% |
| Stroke permanent | 99% |
| New onset dialysis | 99% |
| Sternal infection | 99% |
| CK-MB POD1 | 98% |
| Postoperative peak p-Creatinine | 99% |
| Reoperation for bleeding/tamponade | 99% |
| Reoperation for sternal rupture / deep infection | 99% |
| Reoperation for graft occlusion | 99% |
| Reoperation for valve dysfunction | 99% |
| Reoperation for other reason | 99% |
| Hospital stay time | 100% |
| Discharged from the ward to where | 99% |
| Mortality date | 100% |

BMI: body mass index, CABG: coronary artery bypass graft surgery, CPB: cardiopulmonary bypass, COPD: chronic obstructive pulmonary disease, CK-MB: Creatine kinase-MB isoenzyme, EuroSCORE: European system for cardiac operative risk evaluation, ICU : intensive care unit, LV: left ventricular, NYHA: New York Heart Association functional classification, PAD: pulmonary artery diastolic pressure, POD1: first postoperative day, PHF: postoperative heart failure, RV: right ventricular, SVO_2_: Mixed venous oxygen saturation, VSD: ventricular septal defect.
