# Supplementary material for: Impact of underlying heart disease per se on the utility of preoperative NT-proBNP in adult cardiac surgery
Source: PLoS One. 2018 Feb 8;13(2):e0192503. doi: 10.1371/journal.pone.0192503 (PMC5805306; doi:10.1371/journal.pone.0192503)
Supplement: S2 Table — (DOCX) [file pone.0192503.s002.docx]

**S2 Table.** Perioperative characteristics in all patients with NT-proBNP and those excluded due to missing NT-proBNP values.

|  | All patients  (n=2978) | Patients without preoperative NT-proBNP*  (n=46) | p |
| --- | --- | --- | --- |
| Age (years) | 70 [63-76] | 69 [62-74] | 0.37 |
| Female gender | 24% (714) | 24% (11) | 0.99 |
| Pre-NT-proBNP (ng/L) | 330 [130-970] | - | - |
| BMI (kg/m^2^) | 27 [24-30] | 27 [25-30] | 0.85 |
| Obesity  (BMI≥30kg/m^2^) | 26% (782) | 24% (11) | 0.72 |
| BSA, m^2^ | 1.96 [1.83-2.09] | 1.92 [1.77-2.05] | 0.18 |
| Preop-hemoglobin  (g/L) | 141 [132-150] | 139 [129-145] | 0.09 |
| Preop- creatinine, (µmol/L) | 85 [74-101] | 88 [75-101] | 0.58 |
| Preop-eGFR  (mL•min^-1^ •1.73m^-2^) | 73 [60-86] | 73 [129-145] | 0.94 |
| Preop-albumin  (g/L) | 39 [36-42] | 39 [35-42] | 0.43 |
| Smoker | 11% (336) | 17% (8) | 0.08 |
| Diabetes | 25% (739) | 0 | 0.0001 |
| Hypertension | 65% (1934) | 65% (30) | 0.88 |
| COPD | 7% (219) | 7% (3) | 0.83 |
| Preop Dialysis | 0.8% (25) | 4% (2) | 0.013 |
| Cerebrovascular disease | 7% (208) | 2% (1) | 0.20 |
| Extracardiac arterial disease | 7% (208) | 4% (2) | 0.49 |
| Previous vascular surgery | 4% (115) | 0 | 0.17 |
| Angina | 74% (2207) | 74% (34) | 0.98 |
| Unstable angina | 46%(1372) | 54% (21) | 0.26 |
| CCS IV | 9% (271) | 24% (11) | 0.001 |
| Recent myocardial infarction  (<3 weeks) | 28% (839) | 46% (21) | 0.009 |
| Moderate or severe LV dysfunction | 17% (501) | 17% (8) | 0.92 |
| Severe LV dysfunction | 4% (134) | 9% (4) | 0.18 |
| Preop Pulmonary hypertension | 3% (81) | 0 | 0.26 |
| NYHA III or IV | 58% (1718) | 50% (23) | 0.30 |
| NYHA IV | 8% (239) | 26% (12) | <0.0001 |
| Preop CHF | 17% (506) | 22% (10) | 0.40 |
| Preop atrial fibrillation | 6% (187) | 0 | 0.08 |
| Emergency operation | 3%(88) | 17%(8) | <0.0001 |
| Critical preoperative state | 1% (30) | 7% (3) | 0.0004 |
| Additive EuroSCORE | 4 [3-6] | 5 [3-7] | 0.09 |
| Disease |  |  | 0.37 |
| CAD | 75% (2226) | 80% (37) | 0.38 |
| AS | 14% (406) | 7% (3) | 0.16 |
| MR | 12% (346) | 13% (6) | 0.76 |
| CPB time  (minutes) | 84 [68-104] | 84 [72-112] | 0.43 |
| Aortic cross clamp time (minutes) | 56 [44-70] | 56 [47-68] | 0.91 |
| ICU stay (hours) | 21 [18-23] | 22 [20-38] | 0.010 |
| ICU stay  >72 hours | 6% (192) | 13% (6) | 0.07 |
| Ventilation time (hours) | 3 [2-5] | 4 [3-9] | 0.0007 |
| Ventilation time >48hours | 3% (100) | 11% (5) | 0.006 |
| Severe PHF | 4% (130) | 9% (4) | 0.16 |
| Postoperative stroke | 0.7% (22) | 0 | 0.56 |
| CK-MB POD1 (µg/L) | 16 [10-28] | 20 [13-25] | 0.29 |
| CK-MB POD1  >50 µg•L^-1^ | 12% (345) | 13% (6) | 0.80 |
| Creatinine elevation ≥ 50% | 9% (260) | 9% (4) | 0.98 |
| Postoperative mortality | 2% (53) | 7% (3) | 0.018 |

Data given as medians [interquartile range], percentages (number). AS: aortic stenosis, BMI: body mass index, CAD: coronary artery disease, CHF: congestive heart failure, CPB: cardiopulmonary bypass, COPD: chronic obstructive pulmonary disease, CK-MB: Creatine kinase-MB isoenzyme, eGFR: estimated glomerular filtration rate according to MDRD formula, EuroSCORE: European system for cardiac operative risk evaluation, ICU: intensive care unit, LV: left ventricular, MR: mitral regurgitation, NYHA: New York Heart Association, POD1: first postoperative day, PHF: postoperative heart failure.
