# Supplementary material for: Impact of underlying heart disease per se on the utility of preoperative NT-proBNP in adult cardiac surgery
Source: PLoS One. 2018 Feb 8;13(2):e0192503. doi: 10.1371/journal.pone.0192503 (PMC5805306; doi:10.1371/journal.pone.0192503)
Supplement: S3 Table — (DOCX) [file pone.0192503.s003.docx]

**S3 Table:** Causes of postoperative mortality

| **Patient** | **Cause of death** |
| --- | --- |
| 1 | Heart failure |
| 2 | Fresh MI with acute circulatory failure |
| 3 | Stroke |
| 4 | Unknown |
| 5 | Head trauma / stroke |
| 6 | Stroke |
| 7 | Heart failure |
| 8 | Sternal infection |
| 9 | Heart failure |
| 10 | Heart failure |
| 11 | Gastrointestinal bleeding unspecified acute intestinal ischemia |
| 12 | Infection - myopathy |
| 13 | Heart failure |
| 14 | Heart failure |
| 15 | Heart failure |
| 16 | Sepsis with renal failure |
| 17 | Heart failure |
| 18 | Heart failure after MI |
| 19 | Heart failure |
| 20 | Heart failure |
| 21 | Heart failure |
| 22 | Cardiac arrest, tamponade |
| 23 | Heart failure |
| 24 | Heart failure - MOF |
| 25 | Heart failure |
| 26 | unknown |
| 27 | Heart failure |
| 28 | Heart failure |
| 29 | Renal failure both preoperatively and postoperatively |
| 30 | Heart failure |
| 31 | Heart failure |
| 32 | Heart failure - Renal failure |
| 33 | Respiratory cause |
| 34 | Heart failure after MI |
| 35 | Heart failure - Renal failure |
| 36 | Heart failure |
| 37 | Heart failure |
| 38 | Heart failure |
| 39 | Heart failure |
| 40 | Heart failure |
| 41 | Heart failure |
| 42 | Heart failure |
| 43 | MI, VF cardiac arrest |
| 44 | Heart failure |
| 45 | Heart failure |
| 46 | Heart failure |
| 47 | Heart failure |
| 48 | Heart failure |
| 49 | Heart failure |
| 50 | Heart failure |
| 51 | Heart failure |
| 52 | Heart failure |
| 53 | Heart failure |

Postoperative mortality: mortality within 30 days after cardiac surgery or later during the same hospitalization period including discharge to referral hospital, MI: myocardial infarction, ICU: intensive care unit, MOF: multi-organ failure, VF: ventricular fibrillation.
